# Effective Phenotyping Applications Require Matching Trait and Platform and More Attention to Theory

**DOI:** 10.3389/fpls.2019.01339

**Published:** 2019-10-22

**Authors:** Victor O. Sadras

**Affiliations:** ^1^South Australia Research and Development Institute, Adelaide, South Australia, Australia; ^2^School of Agriculture, Food and Wine, The University of Adelaide, Australia

**Keywords:** Scaling, yield, density-dependence, phenotype, context-dependence

We’re in a maze, not a highway; there is nowhere that speed alone can take us … I’m not being held back by the university’s computers … I’m being held back by my own lack of insight into the problems I’m addressing…

Julie Dehghani

In the spring of 2018, the city of Adelaide hosted the 5th International Plant Phenotyping Symposium (IPPS), titled “From plant, to data, to impact”, as well as the OECD-sponsored workshop “Making science useful to agriculture”^1^. Here I share a personal account of salient findings of these two meetings, with a focus on more effective phenotyping, namely, phenotyping that is more likely to deliver tangible outcomes to plant breeding. To improve the effectiveness of the large phenotyping effort worldwide, I suggest asking two questions:

Are we really limited by data? Or better, to what extent we are limited by data, and to what extent we are, in the terms of Julie Dehghani, being held back by our own lack of insight into the problems we’re addressing? I would argue our theories are lagging, and the theoretical model of the phenotype has become a bottleneck.

Can we improve the matching of trait and phenotyping platform? Individual plants and populations are fundamentally different biological entities; yield is a population-level attribute where plant-plant interactions are important. I would propose that explicit consideration of scaling, density- and context-dependence can help to better match agronomic traits with phenotyping platform and method, avoid expensive distractions and improve return on R&D investment.

The underlying assumption of ‘omics’ technologies is that large datasets on genes, their expression (transcriptomics) and products (proteomics and metabolomics) would resolve the complexities of key traits (Langridge, 2018). These ‘omics’ efforts have been primarily driven by technology—it was possible and therefore done—and largely failed to resolve the agronomically relevant phenotype, particularly crop yield (Langridge, 2018). This failure was ascribed to lagging phenotyping technologies (Cobb et al., 2013; Araus and Cairns, 2014), which in turn motivated the phenotyping effort displayed in the 5th IPPS. The IPPS showed a similar technology-driven approach that leads to large datasets of variable quality and relevance. Once again, we are assuming we are limited by data—but are we? Big data (and associated technologies) is the next promise, and it might deliver in some areas. However, we need hypothesis-driven science at the very least for proper experimental design, and importantly, to match trait and phenotyping platform.

The complete lack of attention to the theoretical aspects of the construction of the phenotype in the 5th IPPS was striking, and reinforces the notion of a technology-driven effort. The oversimplified model based on the unidirectional arrow from genotype to phenotype is unjustified but remains influential (Vinocur and Altman, 2005; Pickett, 2016). More nuanced theories of the phenotype need consideration (West-Eberhard, 2003; Piersma and van Gils, 2011; Noble, 2012; Félix, 2016). The metaphors that genes “control” development, and that genomes embody “programs” for development are particularly misplaced (Noble, 2012; Félix, 2016). A unified theory of phenotypic development and evolution emphasizes that “the individual’s genotype can never be said to control development. Development depends at every step on the pre-existent structure of the phenotype, a structure that is complexly determined by a long history of both genomic and environmental influences” (West-Eberhard, 2003). This perspective is more broadly captured in the concept of downward causation (Noble, 2012; Flack, 2017; Noble, 2017). A panel discussion on what it takes for phenotypic data to be useful focused on technical aspects along the lines of the FAIR principles (Wilkinson et al., 2016). A lonely voice advanced the importance of asking questions that matter biologically and agronomically for data to be useful, but after a lukewarm collective nod the discussion reverted to technical aspects of data management. Future editions of this IPPS will benefit from sessions devoted to the theory of the phenotype.

The 5th IPPS, and the literature more broadly, show that the mismatch between trait and phenotyping approach is a major source of inefficiency, e.g. phenotyping for density-dependent traits such as yield or nutrient uptake in isolated plants in the glasshouse, or in single rows in the field. For different reasons, research in plant biology frequently simplifies to potted plants in chamber or glasshouse. If we are interested in a biological process of an isolated plant in an artificial condition lacking agronomic context, this simplification is fine and needs no justification. However, we must ask rigorous questions about scaling, density- and context-dependence if we want to achieve agronomic relevance.

How does a trait measured in an individual plant relate to the trait in a crop stand, where yield and other agronomic attributes are resolved? Sadras and Richards (2014) classified traits in three groups depending on their scaling. Some traits generally scale, like herbicide tolerance or Bt (**Figure 1A**). There are traits for which scaling is strictly dependent on experimental protocols; for example, leaf expansion rate seems to scale from glasshouse to field in well designed and carefully executed experiments (Reymond et al., 2003). There are traits that rarely scale, including yield (**Figure 1B**), photosynthesis, capture and efficiency in the use of water and nutrients, allometric relationships (**Figure 1C**), and architecture traits such as tillering, shoot and root branching (Sadras and Richards, 2014). Photosynthesis is strongly buffered from molecular to stand level (Pettigrew et al., 1989; Sinclair et al., 2004). Lack of both wind and canopy structure in plant-based settings (glasshouse, chamber) generates unrealistic canopy-atmosphere coupling, hence the difficulty in scaling gas exchange (Jarvis and McNaughton, 1986; Fereres et al., 2014). Artifacts from growing plants in pots are well established (Ben-Porath and Baker, 1990; Passioura, 2002; Passioura, 2006; Poorter et al., 2012; Ryan and Graham, 2018), and yet potted plants are used in phenotyping traits related to water and nutrient uptake and efficiency. Ryan and Graham (2018) critically revised the agronomic relevance of plant-mycorrhiza studies in controlled environments. Density-dependence (Donald, 1963; Harper, 1977) is a useful criterion to predict scaling; tillering responds to neighboring plants as related to both light quality and resources (Casal et al., 1986; Skinner and Simmons, 1993; Peltonen-Sainio and Järvinen, 1995; Robertson et al., 2009), hence is less likely to scale. Nitrogen-biomass allometry—the keystone for quantifying crop nitrogen status (Sadras and Lemaire, 2014; Gastal et al., 2015)—is strongly density dependent, and therefore does not scale from plant to crop (**Figure 1C**). Density-dependence leads to hierarchies in stands and is an artifact commonly overlooked in the glasshouse (Chen et al., 2018) and, to a lesser extent in the field (Rebetzke et al., 2014; Fischer and Rebetzke, 2018). For example, transformed tobacco plants with superior leaf-level photosynthesis were grown with a single-row buffer of shorter, untransformed wild-types leading to a gross misinterpretation of the effect of molecular manipulations on the crop phenotype (South et al., 2019). Gene expression is density-dependent in locust (Pener and Simpson, 2009), and in Arabidopsis (Geisler et al., 2012). In chickpea, Fst genome scan revealed a mismatch in the top genomic regions under selection for yield in border rows under relaxed competition and inner rows under full competition (Lake et al., 2016).

**Figure 1:**
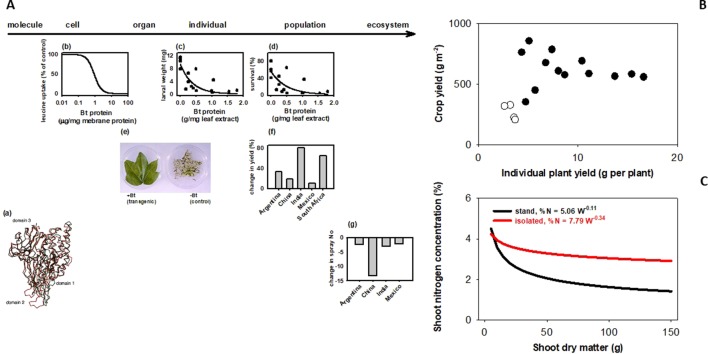
Bt scales, but yield and plant allometry do not because they are density dependent. **(A)** Bt scales from molecular to ecosystem level. (a) *Bacillus turingensis* (Bt) Cry proteins have deleterious effects on Lepidoptera larvae at the (b) cell, (c) individual, and (d) population levels. Bt proteins expressed in transformed plant tissues confer protection at the (e) organ and (f) population level. This protection allows for (g) less reliance on synthetic insecticides for pest control, with consequences at the ecosystem level. Sources: (a) C^a^ traces of individual domains of CryIA(a) (red) and CryIIIA (black) proteins (Grochulski et al., 1995); (b) inhibitory effect of CryIA(a) on leucine uptake by membrane vesicles prepared from *Bombyx mori* larvae midgut; (c) weight and (d) survival of 7-day-old, 3^rd^ instar larvae of *Elsmopalpus lignosellus* after a week of feeding treatments with peanut leaf containing variable concentration of CryIA(c) protein (Singsit et al., 1997); (f) comparison of cotton leaves expressing CryIA(c) proteins and non-transformed controls exposed to *Helicoverpa* spp larva (Lewis Wilson, unpublished); differences between Bt and conventional cotton in (g) lint yield. **(B)** Yield does not scale from plant to population. Yield of wheat stands (200–400 plants m^-2^) does not correlate with yield of isolated plants (<20 plants m^-2^) grown under the same conditions in the field. Source: Pedró et al. (2012). **(C)** Nitrogen-biomass allometry does not scale from plant to population. Owing to plant-plant interactions, dilution curves relating shoot nitrogen concentration and shoot dry matter show a sharp dilution for plants in stands, and a highly buffered dilution in isolated plants. Source: Lemaire and Millard (1999).

Consideration of biological context is critical for effective phenotyping. Environmental factors such as radiation, photoperiod and temperature are spatially and temporally correlated, and plants evolved receptors coupled with molecular signaling that extract information from these joint multivariate properties of the environment. Hence responses dependent on these properties of the environment are encoded in the genome, epigenome and phenome of plants (Karban, 2015). For this reason, phenotyping in an unnatural context, i.e. where correlations between environmental variables have been unrealistically altered, are often of little agronomic relevance because biased relationships among the states of different environmental variables disturb the information decoded by the plant, and hence the phenotype. For example, diurnal profiles of carbon and nitrogen metabolites of plants grown with a step-change in radiation do not match those for plants grown with both regular (day-night sinusoidal cycle) and irregular (due to clouds) fluctuations in radiation (Annunziata et al., 2017). Sowing date trials to screen for heat adaptation in the field bias the relative state of key environmental factors (radiation, photoperiod, temperature, vapor pressure deficit), and confound developmental and thermal-stress responses (Sadras et al., 2015).

The sequence “From plant, to data, to impact” resonates with both the directional “pipeline” in biotechnology, from lab to field (Nuccio et al., 2018) and with the simplistic genotype-to-phenotype model. These directional perspectives have delivered improvements in crop protection but have largely failed to improve yield and adaptation to drought (Dalal et al., 2017; Nuccio et al., 2018). Conceptually, Denis Noble concludes that there is no privileged level of causation (Noble, 2012). Renee Laffite and colleagues have advanced a robust approach that focuses on agronomically rigorous field phenotyping of grain yield and high-level secondary traits such as anthesis-silking interval, with complementary work under controlled conditions—working from field to lab ensures agronomic relevance (Habben et al., 2014; Shi et al., 2015; Brugiere et al., 2017; Lafitte et al., 2018).

To improve the effectiveness of the large phenotyping effort worldwide, I suggest asking two questions:

*Are we really limited by data?* Or better, to what extent we are limited by data, and to what extent we are, in the terms of Julie Dehghani, being held back by our own lack of insight into the problems we’re addressing? I would argue our theories are lagging, and the theoretical model of the phenotype needs attention.

*Can we improve the matching of trait and phenotyping platform*? Individual plants and populations are fundamentally different biological entities; yield is a population attribute. I would propose that explicit consideration of scaling, density- and context-dependence can help to better match agronomic traits with phenotyping platform and method, avoid expensive distractions and improve return on R&D investment.

## Author Contributions

VS wrote the paper.

## Conflict of Interest

The author declares that the research was conducted in the absence of any commercial or financial relationships that could be construed as a potential conflict of interest.

## Footnotes

1 Papers and presentations from this meeting are at: https://msua.aweb.net.au/.
